# Genomic profiles of Japanese patients with vulvar squamous cell carcinoma

**DOI:** 10.1038/s41598-024-63913-z

**Published:** 2024-06-06

**Authors:** Erisa Fujii, Mayumi Kobayashi Kato, Maiko Yamaguchi, Daiki Higuchi, Takafumi Koyama, Masaaki Komatsu, Ryuji Hamamoto, Mitsuya Ishikawa, Tomoyasu Kato, Takashi Kohno, Kouya Shiraishi, Hiroshi Yoshida

**Affiliations:** 1 Division of Genome Biology, National Cancer Center Research Institute, 5-1-1 Tsukiji, Chuo-ku, Tokyo, 104-0045 Japan; 2 Department of Gynecology, National Cancer Center Hospital, Tokyo, Japan; 3 Department of Obstetrics and Gynecology, Juntendo University Faculty of Medicine, Tokyo, Japan; 4 Department of Obstetrics and Gynecology, Showa University School of Medicine, Tokyo, Japan; 5 Department of Experimental Therapeutics, National Cancer Center Hospital, Tokyo, Japan; 6 Division of Medical AI Research and Development, National Cancer Center Research Institute, Tokyo, Japan; 7 Cancer Translational Research Team, RIKEN Center for Advanced Intelligence Project, Tokyo, Japan; 8 Department of Clinical Genomics, National Cancer Center Research Institute, Tokyo, Japan; 9 Department of Diagnostic Pathology, National Cancer Center Hospital, 5-1-1 Tsukiji, Chuo-ku, Tokyo, 104-0045 Japan

**Keywords:** Vulvar squamous cell carcinoma, Genomic profiling, Human papillomavirus, *TP53* mutation, Prognosis, Gynaecological cancer, Cancer genomics

## Abstract

The incidence of vulvar carcinoma varies by race; however, it is a rare disease, and its genomic profiles remain largely unknown. This study examined the characteristics of vulvar squamous cell carcinoma (VSCC) in Japanese patients, focusing on genomic profiles and potential racial disparities. The study included two Japanese groups: the National Cancer Center Hospital (NCCH) group comprised 19 patients diagnosed between 2015 and 2023, and the Center for Cancer Genomics and Advanced Therapeutics group comprised 29 patients diagnosed between 2019 and 2022. Somatic mutations were identified by targeted or panel sequencing, and *TP53* was identified as the most common mutation (52–81%), followed by *HRAS* (7–26%), *CDKN2A* (21–24%), and *PIK3CA* (5–10%). The mutation frequencies, except for *TP53*, were similar to those of Caucasian cohorts. In the NCCH group, 16 patients of HPV-independent tumors were identified by immunohistochemistry and genotyping. Univariate analysis revealed that *TP53*-mutated patients were associated with a poor prognosis (log-rank test, *P* = 0.089). Japanese VSCC mutations resembled those of Caucasian vulvar carcinomas, and *TP53* mutations predicted prognosis regardless of ethnicity. The present findings suggest potential molecular-targeted therapies for select VSCC patients.

## Introduction

The incidence of vulvar carcinoma in Japan is 0.2–0.4 per 100,000 women per year^1^. By contrast, the incidence of vulvar carcinoma is as high as 2.5 per 100,000 women in the USA^2^. A higher prevalence has been reported in South Africa and certain European countries such as Germany, whereas the incidence is low in Western Asia and the Middle East^3,4^. Vulvar squamous cell carcinoma (VSCC) was traditionally considered as a disease affecting postmenopausal women. However, its incidence is increasing among younger people^5^, which is attributed to an increase in the rate of human papillomavirus (HPV) infection^5,6^. HPV is responsible for 22–40% of vulvar carcinomas worldwide^7^. When limited to squamous cell carcinoma, the prevalence of HPV varies from 0 to 86% in different regions, with a pooled prevalence of 39.7%^8^. A study in Japan reported 76% non-HPV VSCC, which may suggest a low prevalence of HPV^9^. A disparity in the prevalence of squamous cell carcinoma between East Asian and Western countries has been identified. Studies in Japan^1,9^ and Korea^10^ reported that approximately 50–60% of vulvar carcinomas are squamous cell carcinomas, which is a lower proportion than that reported by studies in Western countries (75–90%)^7^. These findings suggest that the characteristics of VSCC vary by race and region. However, because of the rarity of vulvar carcinoma and the limited number of Asian population-based studies, the clinicopathological and genomic profiles of VSCC in Asian populations remain unexplored.

The prognosis of VSCC is relatively good for localized disease; however, patients with advanced or recurrent disease have a dismal prognosis. Shin et al. reported a 5-year survival rate of 65.1% for patients with squamous cell carcinoma in Korea^10^, which is comparable with the rates reported in Europe (69.9% in the Netherlands^11^ and 69.9% in the UK^12^). Advances in surgical techniques and chemoradiotherapy have improved the chance of survival for patients with localized disease. However, the prognosis for patients with groin recurrence, distant recurrence, or advanced VSCC has not improved in the past decades, with a reported 5-year survival rate of 25–50%^13^. Clear guidelines for the treatment of recurrent VSCC are lacking^14^, and elucidating the genomic mutational profiles of VSCC is essential to identify potential therapeutic targets.

Recent advances in next-generation sequencing (NGS) have improved our understanding of the characteristics of this rare cancer. Genes that are frequently mutated in VSCC include *TP53* (35–62%), *PIK3CA* (9–35%), *HRAS* (6–14%), and *CDKN2A* (11–38%), although the prevalence of these alterations varies among studies^15–24^. Most studies are based on analysis of Western VSCC patients; therefore, genomic analyses focusing on Asian VSCC patients are needed.

This study investigated the genomic mutational profiles of Japanese patients with VSCC to determine the differences in potential somatic mutations among races and identify somatic mutations related to therapeutic targets or prognosis.

## Results

### Patient characteristics

Nineteen VSCC patients who met the inclusion criteria were enrolled in the NCCH group. The patient characteristics are summarized in Table 1. The age indicates the age at diagnosis. Most of the samples were HPV-independent tumors (84.2%). In this group, all patients with HPV negative tumors had early stage disease. Half of the patients had early-stage disease at the time of diagnosis, whereas the other half had advanced-stage disease. Among the 19 patients, eight experienced recurrence (42.1%) and five died (26.3%).

**Table 1:** Characteristics of patients with vulvar squamous cell carcinoma.

Variables		NCCH	C-CAT
		N = 19 (%)	N = 29 (%)
Age	Median [range]	70 [34–98]	68 [41–86]
HPV status			
p16-IHC	Positive	3 (15.8)	–
	Negative	16 (84.2)	–
PCR-based	Positive	3 (15.8)	–
	HPV genotype (16/52/58)	1/1/1	
	Unknown	–	29 (100)
Stage (FIGO 2021)	I	8 (42.1)	–
	II	1 (5.3)	–
	III	9 (47.3)	–
	IV	1 (5.3)	–
	Unknown	–	29 (100)
Surgery	Vulvectomy	18 (94.7)	–
	Unknown	–	29 (100)
Neo and adjuvant therapy	None	12 (63.2)	–
	Radiotherapy	6 (31.5)	–
	CCRT	1 (5.3)	–
	Unknown	–	29 (100)
Recurrence	Yes	9 (47.3)	28 (96.6)
Follow-up time (months)	Median [range]	14.5 [7–61]	–
Clinical outcome	Alive	14 (73.7)	15 (51.7)
	Dead	5 (26.3)	10 (34.5)
	Unknown	–	4 (13.8)

NCCH, National Cancer Center Hospital; C-CAT, Center for Cancer Genomics and Advanced Therapeutics; HPV, human papilloma virus; IHC, Immunohistochemistry; PCR, polymerase chain reaction; FIGO, The International Federation of Gynecology and Obstetrics; CCRT, concurrent chemoradiation therapy.

The age indicates the age at diagnosis.

The clinical characteristics of the 29 patients in the C-CAT group are also summarized in Table 1. The age also indicates the age at diagnosis. Because C-CAT collects data from patients with advanced or progressive disease, most cases were relapsed (96%).

Of the 19 cases, p53 IHC was performed in 16. All three HPV-associated tumors exhibited wild-type staining patterns. In comparison, in 13 cases considered HPV-independent tumors, diffuse strong positive staining was observed in eight cases, and a wild-type pattern was observed in five cases (Supplementary Table 1).

Among 19 cases, three were classified as HPV-associated tumors based on p16 block-type positivity, and 13 were classified as HPV-independent. Regarding preinvasive lesions, classic vulvar intraepithelial neoplasia (VIN) 3 was observed in three cases of HPV-associated tumors. In comparison, differentiated VIN was observed in nine cases of HPV-independent tumors, and differentiated exophytic vulvar intraepithelial lesions (DE-VIL) were observed in two cases. Lymphovascular invasion was observed in 12 of 18 cases. Lymph node dissection was performed in 16 of the 19 cases, with a median number of excised lymph nodes of 14 (range, 1–56). Lymph node metastasis was observed in 10 of 17 evaluable cases, with three cases showing extracapsular invasion of the lymph nodes. Surgical margins were evaluated in 18 primary tumor resection cases, excluding one lymph node metastasis resection, with 13 cases showing negative margins, five positive horizontal margins, and one positive vertical margin (Supplementary Table 1).

### Genomic alterations in VSCC detected in the two groups

**Figure 1:**
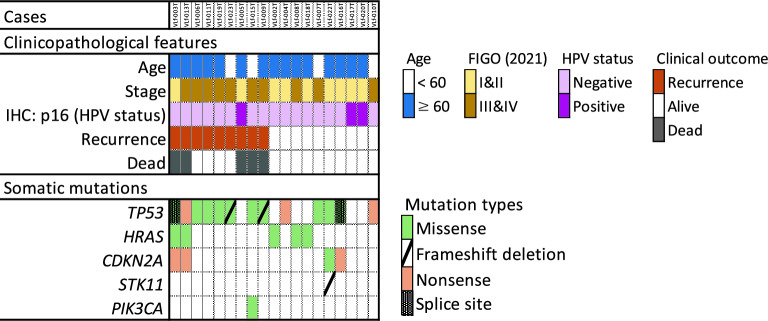
Clinicopathological characteristics and somatic mutations in the NCCH group. The 19 patients are categorized according to clinicopathological features and major somatic mutated genes. Mutated genes are color-coded according to mutation type.

In the NCCH group, mutation patterns were observed in all HPV-independent tumors, whereas no mutations were found in HPV-dependent tumors (Fig. 1). The gene with the highest frequency of somatic mutations was *TP53* (81%), followed by *HRAS* (26%), *CDKN2A* (21%), and *PIK3CA* (5%) (Fig. 1 and Supplementary Table 2). The rate of *TP53* mutations in recurrent cases was remarkably high (89%). The candidate somatic mutations in the two groups are listed in Supplementary Tables 2 and 3. In both groups, most *TP53* mutations occurred at the DNA-binding domain (DBD), a 191-amino-acid protease-resistant fragment (residues 102–292) containing hotspot mutations at R175, R248, R273, and R282. Most *TP53* mutations are located in the DBD^25^, and the mutation profile was similar to those previously reported for VSCC.

**Figure 2:**
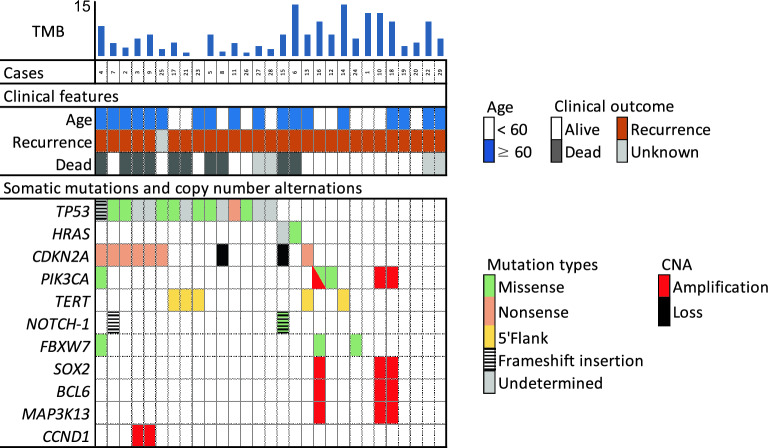
Clinical features and genomic alterations in the C-CAT group. The 29 patients are categorized according to available clinical data and major somatic mutated genes. Mutated genes are color-coded according to mutation type.

In the C-CAT group, *TP53* was also the most frequently mutated gene (52%), followed by *CDKN2A* (24%), *PIK3CA* (10%), and *HRAS* (7%) (Fig. 2 and Supplementary Table 3). In the C-CAT and NCCH groups, *TP53* mutations were detected in > 50% of patients, showing a trend toward a higher mutation proportion than previously reported (Table 2). The frequency and type of mutations in other genes were similar to those observed in Western populations. Regarding copy number alterations, *PIK3CA*, *SOX2*, *BLC6*, *and MAP3K13* amplification was detected in three patients (10%) and *CCND1* alterations were observed in two patients (7%). *CDKN2A* loss was detected in two different cases (7%).

**Table 2:** Frequency of mutations in top mutated genes from NCCH, C-CAT, and previous studies.

Study	N	Platform	Mutated gene (%)			
			*TP53*	*HRAS*	*CDKN2A*	*PIK3CA*
NCCH	19	Ion AmpliSeq™ Cancer Hotspot Panel v2	81	26	21	5
C-CAT	29	FoundationOne® CDx/OncoGuide™ NCC Oncopanel	52	7	24	17
Zięba et al.^15^	81	Ion AmpliSeq™ Cancer Hotspot Panel v2	44	6	23	9
Salama et al.^16^	28	Memorial sloan kettering‐integrated mutation Profiling of actionable cancer targets (MSK‐IMPACT™)	62	N/A	38	35
Weberpals et al.^17^	43	Ion AmpliSeq™ Cancer Hotspot Panel v2	35	14	11	23

NCCH, National Cancer Center Hospital; C-CAT, Center for Cancer Genomics and Advanced Therapeutics; N/A, not applicable.

The gene mutation frequencies from previously reported genomic analyses of VSCC are summarized in Table 2. The most commonly observed genomic alterations in these studies were in *TP53* (35–62%), *PIK3CA* (9–35%), *CDKN2A* (11–38%), and *HRAS* (6–14%).

### *TP53* mutation as a prognostic factor

**Figure 3:**
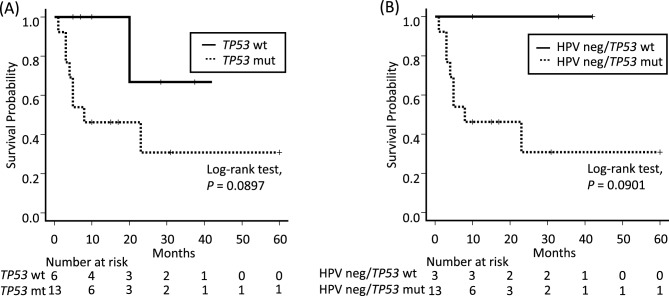
Correlation between *TP53* gene mutational status and recurrence-free survival in the NCCH group. (**A**) Kaplan–Meier survival curves showing the clinical outcomes of 19 patients. (**B**) Kaplan–Meier survival curves show the clinical outcomes of 16 HPV-independent patients. *TP53* wt = *TP 53* wild-type; *TP53* mt = *TP53* mutated; HPV neg/*TP53* wt = HPV-independent/*TP53* wild-type; HPV neg/*TP53* mt = HPV-independent/*TP53* mutated.

In the NCCH group, the frequency of *TP53* mutations was 8/9 in patients with recurrent disease and 5/9 in patients without recurrent disease. *TP53* mutation correlated with a worse prognosis regarding relapse-free survival (log-rank test, *P* = 0.089) (Fig. 3A), and this association was also observed in patients without HPV infection (log-rank test, *P* = 0.090) (Fig. 3B). However, did not correlate with worse overall survival in the NCCH and C-CAT groups (log-rank test, *P* = 0.45, *P* = 0.13) (Fig. S3).

## Discussion

In this study, we investigated the genomic mutational profiles of 48 Japanese VSCC patients and compared them with those of Caucasian patients with VSCC data obtained from previous genomic analyses. The mutational pattern was comparable between Japanese and Caucasian VSCC patients. *TP53* was the most frequently mutated gene, followed by *HRAS*, *CDKN2A*, and *PIK3CA*. *TP53* mutation correlated with clinical outcome, which supports previous studies identifying *TP53* mutation as a prognostic factor.

The frequency of common gene mutations in the present groups did not deviate significantly from that reported previously, except for *TP53* mutations, which were observed more frequently. *TP53*, a crucial tumor suppressor gene, is the most frequently mutated gene in human cancers^26^. Corey et al. detected *TP53* mutations in approximately 80% of VSCCs without HPV infection^24^, which is consistent with the present findings. Racial disparity regarding the frequency of *TP53* mutations in VSCC may be due to differences in the prevalence of HPV infection. Kang et al.^7^ suggested that there is geographical variation in HPV prevalence in vulvar carcinoma. The HPV infection rate in the present group was 16%, whereas that reported previously is approximately 40%^8^. Therefore, the higher frequency of *TP53* mutations in this group may be due to the low proportion of HPV-associated VSCC, which is characterized by a low frequency of *TP53* mutation. The present findings suggest that genetic factors alone do not account for the regional variation in the epidemiology of vulvar carcinoma.

*TP53* mutation is significantly associated with advanced disease and a poor prognosis for various cancer types^27,28^. In this group, the recurrence rate was notably higher in cases with *TP53* mutations. Although the number of VSCC patients analyzed was not sufficient to establish *TP53* alteration as a poor prognostic factor, the correlation between *TP53* alterations and clinical outcomes in VSCC was examined in previous studies (Table 3)^18–22^. In previous studies, *TP53* alterations were detected used Sanger sequencing, targeted panel sequencing, and IHC. Nooij et al.^18^ reported worse recurrence-free survival in an HPV negative/p53 mutated group (*P* = 0.044) when comparing three groups based on *TP53* mutational status and HPV status (i.e., HPV positive, HPV negative/p53 wildtype, and HPV negative/p53 mutated groups). Kortekaas et al.^19^ and Lérias et al.^20^ also showed significant differences in recurrence-free survival (*P* < 0.001) and rate of recurrence (*P* = 0.0005) according to *TP53* mutation status. By contrast, Tessier-Cloutier et al.^22^ showed that *TP53* mutation alone was not an independent prognostic factor (*P* = 0.23 for OS and *P* = 0.35 for PFS), whereas *TP53* and *PIK3CA* double mutation was associated with a poor prognosis (*P* = 0.0016 for OS and *P* = 0.034 for PFS). Choschzick et al.^21^ did not identify *TP53* as an independent prognostic factor (*P* > 0.05 for cumulative survival) when comparing three groups with different *TP53* mutation and HPV status. In brief, although *TP53* mutation correlates with worse prognosis in several studies^19–21^, other studies report opposite findings^22,23^. *TP53* mutation may be a poor prognostic factor for VSCC, although further studies are needed to confirm the role of *TP53* mutation as a critical predictor of worse clinical outcomes.

**Table 3:** Correlation between *TP53* status and clinical outcomes in previous vulvar squamous cell carcinoma studies.

Study	Region of study population	Number of subjects	NGS or IHC	Log-rank test	Clinical outcomes	Compared groups
Nooij et al.^19^	Netherlands	236	Panel (VC NGS panel)	*P* = 0.044	Recurrence-free survival	**HPV−/p53mut** vs. HPV−/p53wt vs. HPV+
Tessier-Cloutier et al.^23^	Canada	34	Panel (targeted sequence)	*P* = 0.35	Progression-free survival	HPV−/p53wt vs. HPV−/p53mut
Choschzick et al.^22^	Germany	25	Sanger sequence	*P* > 0.05	Cumulative survival	**HPV+/p53wt** vs. HPV−/p53mut vs. HPV−/p53wt
Kortekaas et al.^20^	Netherlands	413	IHC	*P* < 0.001	Recurrence-free period	**HPV−/p53abn** vs. HPV+
Lérias et al.^21^	Portugal	84	IHC	*P* = 0.0005	Rate of recurrence	**p53abn** vs. p53wt

NGS, next generation sequencing; IHC, immunohistochemistry.

Worst prognosis group is shown in bold.

In VSCC, *TP53* was the most frequently mutated gene, followed by *HRAS*, *CDKN2A*, and *PIK3CA,* regardless of ethnicity. *TP53* mutations, which are present in many cancer types, are considered targets for novel cancer therapies^28–30^. A recent study by Guiley^31^ reported compounds that directly target mutant p53; however, further studies will be required to determine whether these compounds are therapeutically effective. *HRAS*, a member of the RAS family of genes, is also known as the mouse sarcoma virus and is associated with human cancer. Tipifarnib is a farnesyltransferase inhibitor that suppresses *HRAS* function and is used as a targeted therapy for *HRAS*-mutated tumors^26,32, 33^. Ho et al. investigated the efficacy of tipifarnib in head and neck squamous cell carcinoma patients with a mutation in *HRAS*^34^, reporting an objective response rate of 55%. Loss of the tumor suppressor gene *CDKN2A*, which encodes p16 and p14, is a frequent occurrence in VSCC. Hsu et al. reported that CDK4/6 inhibitors are a breakthrough therapy for a subset of sarcomas^35^. Therapeutic strategies targeting *CDKN2A* loss hold great potential for the treatment of various cancer types including VSCC^36^. Pharmacological inhibition of the p16 targets CDK4/6 is a prime example of such a strategy^37^, although additional studies are needed prior to its clinical application. BYL-719 is a PIK3CA inhibitor with low drug-drug interactions, suggesting its potential for combinatorial therapy of recurrent/advanced cervical cancer^38^. Alpelisib (BYL-719) plus fulvestrant was recently approved for the treatment of ER-positive breast cancer patients who develop resistance to ER-targeted therapy^39^. An expansion in the indications for molecular-targeted drugs for VSCC is expected.

This study had several limitations. The number of patients was limited because of the rarity of this cancer. Some clinical data in the C-CAT group were not available, and further validation studies are needed. Additionally, because genomic testing requires a certain amount of tissue, early-stage disease cases and small tumors were not included in the analysis, which may have led to selection bias in the NCCH group.

In conclusion, we found similarities in the somatic mutation profiles of VSCC between Japanese and Caucasian populations. This suggests that the genetic etiology of VSCC does not differ significantly according to ethnic background. Analysis of the correlation between *TP53* mutation status and clinical outcomes confirmed previous findings identifying *TP53* mutation as a prognostic factor. Molecular-targeted therapies may be indicated for selected VSCC patients based on somatic mutational profiles. This study, which included a specific number of Asian patients, will contribute to our understanding of this less investigated yet clinically crucial disease.

## Materials and methods

### Characteristics of 48 patients with VSCC (divided into two groups)

The clinical data used in this study were obtained retrospectively from patient records. The study included 48 Japanese patients with primary VSCC who were divided into two independent groups. The decision trees for patient selection are provided in Fig. S1. Forty-two vulvar carcinoma patients were treated at the National Cancer Center Hospital (NCCH) between January 2015 and January 2023. The tumors were diagnosed pathologically according to the 2021 International Federation of Gynecology and Obstetrics criteria, and classified according to the World Health Organization classification of tumors. Of the 42 patients, 23 were excluded due to the histological type or insufficient material; therefore, 19 patients were eligible for inclusion in the NCCH group. Among the 91 patients who were diagnosed between 2019 and 2022 and registered at the Center for Cancer Genomics and Advanced Therapeutics (C-CAT) databank (ver. 20221218), 30 patients with vaginal cancer and 32 patients with other histological types were excluded. Ultimately, 29 patients with VSCC were included in the C-CAT group. In this study, there was no overlap between patients in the NCCH group and those in the C-CAT group. C-CAT is a national data center for cancer genomic medicine in Japan that was established in 2019^40^. Comprehensive genomic profiling tests used were FoundationOne^®^ CDx and OncoGuide™ NCC Oncopanel. As of June 30, 2022, C-CAT has aggregated 36,340 data points of clinical/genomic information, and is open for sharing with academic institutions and industries.

This study was performed in accordance with the Declaration of Helsinki and approved by the institutional review board of the National Cancer Center Research Institute (approval numbers 2017-136, 2020-067) and the C-CAT Data Utilization Review Board (approval number CDU2021-001N). All participants provided written informed consent.

### Immunohistochemistry (IHC) of p16

IHC was performed using p16 as a surrogate marker to identify HPV-associated tumors. H&E (Fig. S2A and C) and IHC staining were performed on representative whole sections of tumor from formalin-fixed, paraffin-embedded (FFPE) specimens, and the paraffin blocks were sectioned at a thickness of 4 μm. IHC was performed using a Ventana Benchmark Ultra automated immunostainer (Tucson, AZ, USA) and a mouse monoclonal antibody specific for p16 (E6H4, prediluted, Roche diagnostics) according to standard protocols and with appropriate positive and negative controls. A case was classified as an HPV-associated tumor only if it showed block-positive p16 staining, with diffuse nuclear or nuclear and cytoplasmic positivity (Fig. S2B). Negative results (Fig. S2D) were defined as total absence of staining or weak, focal, and discontinuous staining, which indicated HPV-independent tumors.

The results of p53 immunohistochemical staining analyses were available for 16 out of 19 cases. Staining was performed using a p53 monoclonal antibody (DO7, prediluted, Agilent/Dako, Glostrup, Denmark) in all cases.

### DNA preparation and targeted sequencing

FFPE tissue blocks from a VSCC sample were retrieved and serially sectioned at 10 μm thickness on slides. The slides were deparaffinized, and the H&E-stained sections were used for macrodissection. Genomic DNA was extracted from 19 VSCC tissue samples using the QIAamp DNA FFPE tissue kit according to the manufacturer's instructions (Qiagen, Hilden, Germany). Genomic DNA (50 ng) purified from tumor tissues was used for library construction using the Ion AmpliSeq™ Cancer Hotspot Panel v2 (Thermo Fisher Scientific, Waltham, MA, USA). Sequencing was performed on the Ion Proton platform (Thermo Fisher Scientific). For quality control, samples with a mean read depth of coverage > 1000 and a base quality score of 20 (with < 1% probability of being incorrect), which accounted for 90% of total reads, were selected^41–44^.

Data were analyzed using Torrent Suite Software v5.0.4 (Thermo Fisher Scientific). First, somatic mutations were selected using the following criteria: (i) variant allele frequency of somatic mutations in tumor tissues > 4%; (ii) removal of single nucleotide polymorphisms if they showed a threshold allele frequency of ≥ 0.01 in either the NHLBI GO Exome Sequencing Project (ESP6500) (http://evs.gs.washington.edu/EVS/) or the Japanese Multi Omics Reference Panel (jMorp, 20210907) (https://jmorp.megabank.tohoku.ac.jp/ijgvd/)^45^; and (iii) registration of mutations as “pathogenic/likely pathogenic variants” in ClinVar^46^ or “oncogenic/likely oncogenic mutations” in OncoKB^9^. Finally, the selected variants were analyzed by manual inspection using the Integrative Genomics Viewer (IGV; http://www.broadinstitute.org/igv/)^47^.

### HPV genotyping by Sanger sequencing

The 19 samples were subjected to HPV genotyping. Genomic DNA (10 ng) was amplified by PCR targeting two distinct HPV genomic regions using TaKaRa Taq DNA polymerase (Takara Bio Inc., Shiga, Japan). The PCR reaction was performed as described previously^41^. Sanger sequencing was performed using an ABI 3130xl DNA Sequencer (Applied Biosystems). The similarity between the obtained sequences and various HPV genotypes in the GenBank database was determined using the Basic Local Alignment Search Tool (BLAST) (https://blast.ncbi.nlm.nih.gov/Blast.cgi).

### Statistical analysis

Statistical analysis was performed using EZR version 1.61 (Saitama Medical Center, Jichi Medical University, Saitama, Japan), a graphical user interface for R (The R Foundation for Statistical Computing, Vienna, Austria)^48^. The level of statistical significance was set at *P* < 0.05. Cumulative survival was estimated using the Kaplan–Meier method, and differences in survival between groups were analyzed using the log-rank test.

### Supplementary Information

Supplementary Figure 1.

Supplementary Figure 2.

Supplementary Figure 3.

Supplementary Legends.

Supplementary Tables.

## Data Availability

The C-CAT dataset used in this study was obtained from the C-CAT database (https://www.ncc.go.jp/jp/c_cat/use/index.html). The datasets used and analyzed during this study are available from the corresponding author upon reasonable request.
