# Supplementary material for: Genomic profiles of Japanese patients with vulvar squamous cell carcinoma
Source: Sci Rep. 2024 Jun 6;14:13058. doi: 10.1038/s41598-024-63913-z (PMC11156893; doi:10.1038/s41598-024-63913-z)
Supplement: Supplementary file 1 — Supplementary Figure 1. [file 41598_2024_63913_MOESM1_ESM.ppt]

## Slide 1
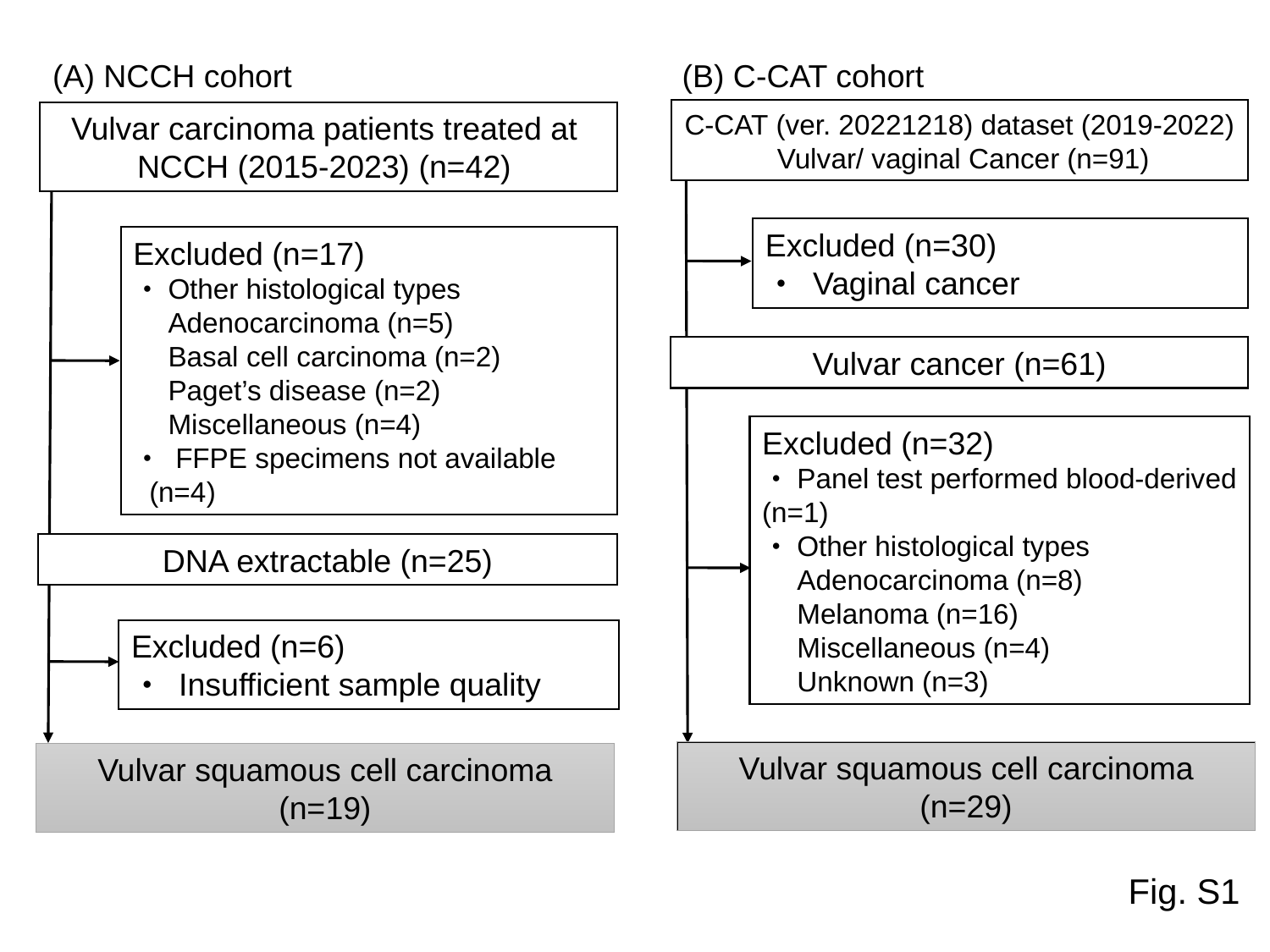

(A) NCCH cohort
Vulvar carcinoma patients treated at NCCH (2015-2023) (n=42)
Excluded (n=17)
・Other histological types
　Adenocarcinoma (n=5)
　Basal cell carcinoma (n=2)
　Paget’s disease (n=2)
　Miscellaneous (n=4)
・ FFPE specimens not available (n=4)
DNA extractable (n=25)
Excluded (n=6)
・ Insufficient sample quality
Vulvar squamous cell carcinoma (n=19)
(B) C-CAT cohort
C-CAT (ver. 20221218) dataset (2019-2022)
 Vulvar/ vaginal Cancer (n=91)
Excluded (n=30)
・ Vaginal cancer
Vulvar cancer (n=61)
Excluded (n=32)
・Panel test performed blood-derived (n=1)
・Other histological types
　Adenocarcinoma (n=8)
　Melanoma (n=16)
　Miscellaneous (n=4)
　Unknown (n=3)
Vulvar squamous cell carcinoma (n=29)
Fig. S1
