# Supplementary figures and images for: Genomic profiles of Japanese patients with vulvar squamous cell carcinoma
Source: Sci Rep. 2024 Jun 6;14:13058. doi: 10.1038/s41598-024-63913-z (PMC11156893; doi:10.1038/s41598-024-63913-z)

## Slide 1
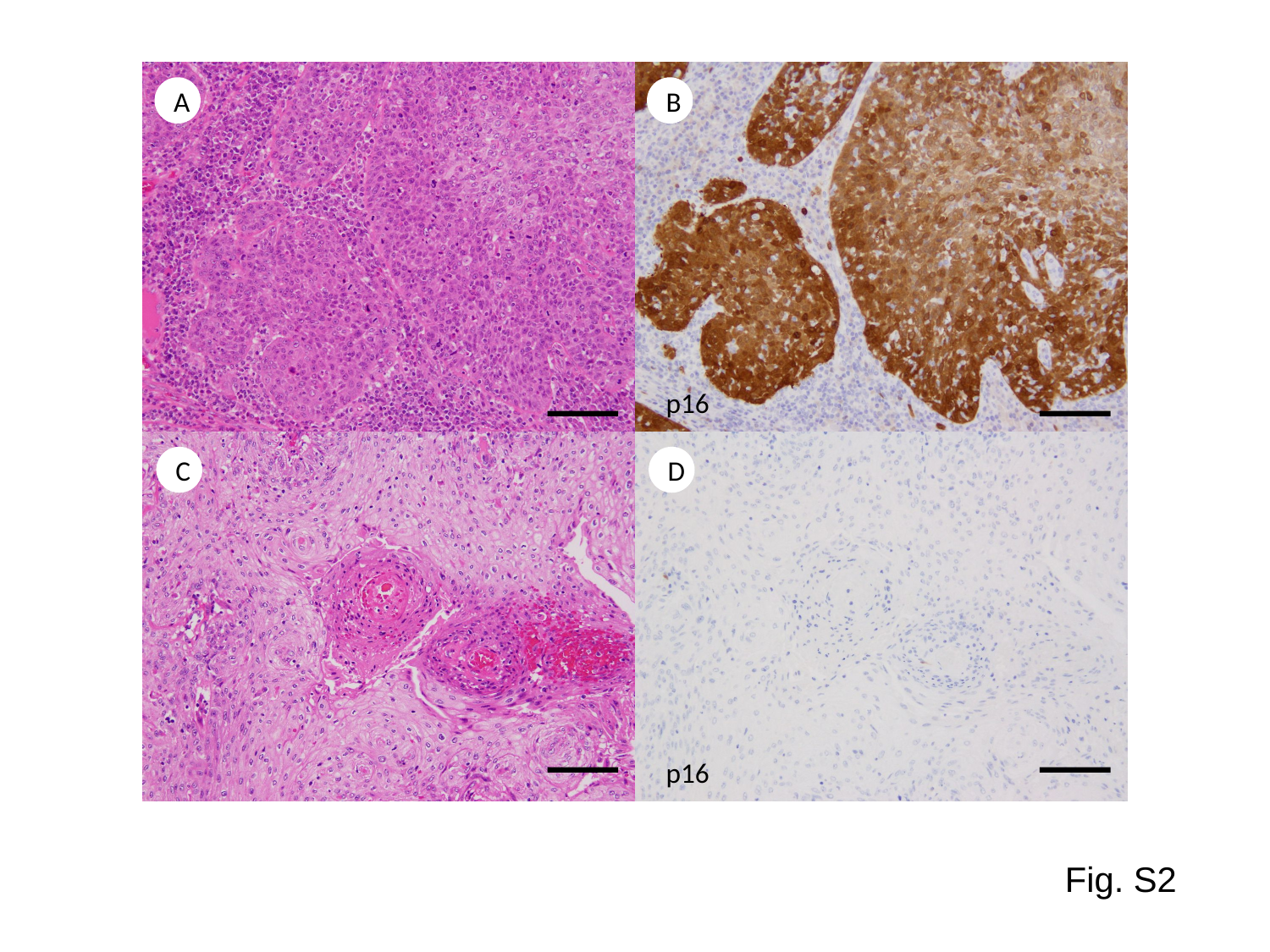

A
B
p16
C
D
p16
Fig. S2

Supplement: Supplementary file 2 — Supplementary Figure 2. [file 41598_2024_63913_MOESM2_ESM.ppt]

## Slide 1
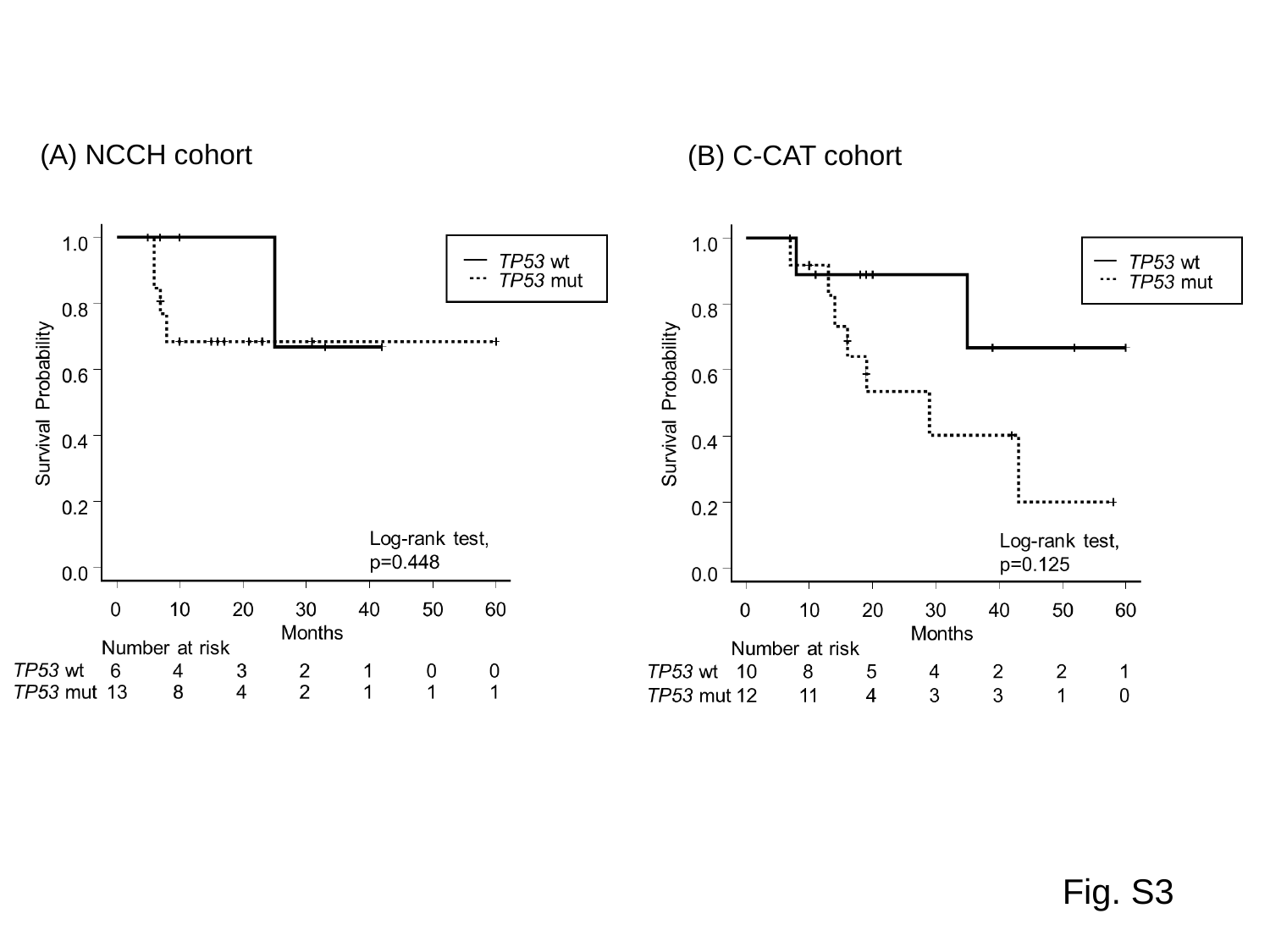

(A) NCCH cohort
(B) C-CAT cohort
Fig. S3

Supplement: Supplementary file 3 — Supplementary Figure 3. [file 41598_2024_63913_MOESM3_ESM.ppt]
