# Supplementary material for: Genomic profiles of Japanese patients with vulvar squamous cell carcinoma
Source: Sci Rep. 2024 Jun 6;14:13058. doi: 10.1038/s41598-024-63913-z (PMC11156893; doi:10.1038/s41598-024-63913-z)
Supplement: Supplementary file 4 — Supplementary Legends. [file 41598_2024_63913_MOESM4_ESM.doc]

**Supplementary Information**

**Genomic profiles of Japanese patients with vulvar squamous cell carcinoma**

**Fujii E et al.**

**Supplementary Figure legends.**

**Figure S1. Patient selection.**

Patient selection flowchart for the NCCH group (A) and the C-CAT group (B).

**Figure S2. Representative microphotographs of p16 immunohistochemistry.**

(A) HPV-associated squamous cell carcinoma of the vulva (H&E, x200). The tumor cells show block-type positivity of p16 (B, x200). (C) HPV-independent squamous cell carcinoma of the vulva (H&E, x200). The tumor cells are negative for p16 (D, x200). Black bars (A-D) indicate 100 μm.

**Figure S3. Correlation between *TP53* gene mutational status and overall survival.**

(A) Kaplan–Meier survival curves showing the clinical outcomes of the NCCH patient group. (B) Kaplan–Meier survival curves showing the clinical outcomes of the C-CAT patient group.

*TP53* wt = *TP 53* wildtype, *TP53* mt = *TP53* mutated.
